# Education and socioeconomic status as predictors of refugee mental health: insights from a study of Jordan-based Syrian refugee sample

**DOI:** 10.3389/fpubh.2024.1432205

**Published:** 2024-10-09

**Authors:** Fatin Atrooz, Omar F. Khabour, Fidaa Almomani, Sally Aljararwah, Batool H. Alfurjani, Samina Salim

**Affiliations:** ^1^Department of Pharmacological and Pharmaceutical Sciences, College of Pharmacy, University of Houston, Houston, TX, United States; ^2^Department of Medical Laboratory Sciences, Faculty of Applied Medical Sciences, Jordan University of Science and Technology, Irbid, Jordan; ^3^Department of Rehabilitation Sciences, Faculty of Applied Medical Sciences, Jordan University of Science and Technology, Irbid, Jordan

**Keywords:** health disparities, university education, mental health, Syrian refugees, Jordan

## Abstract

**Background:**

The Syrian civil war is considered as the greatest humanitarian crisis in modern history, which has resulted in a major refugee crisis. A significant concern is the high prevalence of mental disorders such as depression, anxiety, and post-traumatic stress disorder (PTSD) among Syrian refugees. While the focus of most refugee mental health research has been pre-migration trauma, post-migration challenges and stressors, which can also be significant determinants of mental health, are often ignored. The purpose of this study was to assess mental health of Syrian refugees as compared to local Jordanians, and to examine sociodemographic factors and perceived stressors that are associated with mental distress among participants.

**Methods:**

This was a cross sectional study conducted in northern region of Jordan among Syrian refugee and Jordanian adults. We used the validated Arabic version of Afghan Symptoms Checklist (ASC) to assess mental distress among participants.

**Results:**

A total of 929 subjects (43% Syrian refugees, 56% females) participated in this study. Disparities in education, monthly income, and health insurance were significant between Syrian refugees and Jordanians, *p* < 0.001. The mean score in ASC was significantly higher among Syrian refugees, particularly among refugee females (mean ASC score ± standard deviation: Syrian refugee females: 58.22 ± 1.13; Syrian refugee males: 45.31 ± 1.28; Jordanian females: 51.06 ± 0.91; Jordanian males: 46.45 ± 1.08, *p* = 0.002). Multivariable linear regression showed that the estimated difference in the mean of ASC score between males and females is 7.42 (*p* < 0.001), and the estimated mean difference between Syrian refugees and Jordanians is 2.76 (*p* = 0.019). Interestingly, the estimated mean of ASC scores for individuals with high education level is decreased by 4.18 as compared to individuals with lower education level (high school or less), *p* < 0.001.

**Conclusion:**

Education level was highly predictable of mental distress of Syrian refugees, particularly female refugees. Enhancing higher educational opportunities is proposed for improving socioeconomic status of refugees which might serve as a buffering strategy for mental distress among this vulnerable population.

## 1 Introduction

The Syrian refugee crisis has put many countries under considerable pressure to accommodate and integrate large numbers of refugees, especially neighboring countries like Jordan, Lebanon, and Turkey. According to the World Bank classification system, Jordan is an upper-middle income country with high economic vulnerability due to the refugee crises, currently compounded with the most recent Israel-Hamas war and the climate change-associated water crises. Considering Jordan’s limited resources and its geopolitical environment, the Kingdom faces many challenges which impact Jordanian as well as the refugee communities. An estimated 1.4 million Syrian refugees live in Jordan with more than 80% of them residing in the urban communities, while the remaining living in the refugee camps (47). The majority of Syrian refugees live below the poverty line with limited health insurance and minimal access to medical care, hence they continue to be at a great risk of diseases (1). A significant concern is the mental health status of this displaced group. In general, the global mental health burden of refugees is well documented (2). A recent meta-analysis revealed that the prevalence of post-traumatic stress disorder (PTSD) and depression among refugees was highly significant with a 31.46% prevalence of PTSD and 31.5% prevalence of depression (3) as compared to the prevalence of PTSD and depression in the general population (3.9 and 4.4%, respectively) (4). Importantly, existing evidence also indicates that the refugees continue to remain highly vulnerable to mental distress for several years after they have resettled in host countries (2). This vulnerability arises due to various risk factors for mental disorders that refugees continue to encounter throughout their resettlement processes, including before, during, and after displacement and migration (5). The United Nations High Commissioner for Refugees (UNHCR), the World Health Organization (WHO), non-profit organizations, mainstream mental health and specialist refugee services are tasked to support mental health of refugees, but the reality is that financial needs take precedence and the mental health needs of most refugees takes a back seat, with most refugees never being evaluated for mental health problems or ending up not receiving appropriate services even when deemed mentally ill. The primary reasons being scarcity of resources, inequitable distribution of services, barriers to accessing medical care even when services are available, and stigma associated with being both a refugee and mentally ill. Importantly, in our recently published study, Syrian refugee women resettled in an urban area of Northern Jordan, reported significantly high perceived stress and mental distress as compared to local Jordanian women (6). Factors driving these stressed conditions remain unclear and is the focus of the present study. It is important to identify predictors of mental stress such that full blown mental disorders are averted from developing and better mental health outcomes are achieved.

Historically, the focus of mental health research and targeted interventions for refugees has been pre-migration trauma, which may be a good predictor of mental disorders such as PTSD, but the post-migration context, as it relates to the structural challenges such as education, and employment can also be a significant determinant of mental health. These post-migration challenges often do not get the attention this deserves. For example, Syrian families resettling in Lebanon reportedly faced limited access to health care, employment and education (7–9), children reported discrimination, harassment and social isolation (10), and young refugee boys reported physical violence and exploitation as child laborers (10) while girls reported having experienced gender-based violence and forced marriage or child labor (11, 12). Thus post-migration factors can be consequential for mental health of refugees, and more information must be gathered to be able to comprehensively address these structural challenges faced by refugees in host countries. Clearly, there is a paucity of research on social and material factors of displacement (13). This is a missed opportunity as post-migration factors including structural challenges such as education and employment, two factors that are potentially critical to the mental health of this vulnerable group must be addressed (12, 14). Importantly, there is evidence to suggest that education and employment have the potential to moderate the ability of refugees to recover from pre-migration trauma. For example, refugees whose mental health improved by 8–9-year follow-up were more likely to be educated and/or employed than were those who remained symptomatic (15).

Furthermore, post-migration stressors can be quite consequential for refugee mental health, hence there is a critical need for devising targeted theory-based interventions to address refugee mental health needs (16). This can be achieved by collecting psychosocial information and incorporating this knowledge with the social determinants of health in assessing comprehensive health risks for refugees in a broader context of social, economic and political climate (17, 18). Relevant to this, low socioeconomic status (SES) which is defined as low education level or low income is considered as an important predictor of increased mental distress (19). Prior research has documented a strong and positive correlation between completed education and adult mental health (20). Researchers often describe this important relationship using causal expression: higher levels of education are thought to enhance individual’s skills, afford important structural advantages, and empower better coping mechanisms, which all lead to better mental health (21).

Despite the importance of higher education for refugee well-being and mental health, access to higher education is severely limited with only 7% of refugees worldwide reportedly attending universities (48). According to the Jordanian Planning Ministry, most of the Syrians who arrived Jordan after the civil war are registered as refugees, giving the country the second-highest *per capita* share of Syrians in the world. International donors have stepped in to mitigate the socioeconomic challenges arising from hosting long-term displaced populations. Relief groups and their supporters have prioritized providing primary school education, as most donors see higher education for refugees as a luxury option. With few resources and a sporadic, short-sighted approach to funding has left university degrees out of reach for most Syrian students. Only 3% of Syrian refugees in Jordan pursue higher education which is due to a complex array of factors, including lack of identity documentation and proof of former study, institutional policy restrictions, financial hardship, and the limited capacity of Jordanian higher education institutions to accept large numbers of refugee students (22). The goal of the present study, was to test the hypothesis that low education level is a predictor of mental distress among Syrian refugee communities, particularly among female refugees.

## 2 Methods

This is a cross sectional study conducted in northern urban parts of Jordan between October 2022–July 2023. The study was approved by the Institutional Review Board (IRB) Committee for the Protection of Human Subjects, University of Houston (UH), Houston, TX, US (STUDY00002929) and by the Jordan University of Science and Technology (JUST) IRB, Irbid, Jordan (IRB#52/148/2022, 10.05.2022).

### 2.1 Subject recruitment

Following study approval from UH-IRB and JUST-IRB Committees, Syrian refugees resettled in northern Jordan and Jordanian men and women living in the same urban areas were recruited through JUST students enrolled in Master’s program using convenient and snowball sampling methods. The basic criteria of inclusion were adult Syrian refugee or Jordanian women and men (18 years or older). Individuals with self-reported history of Schizophrenia, Parkinson’s disease, or Alzheimer’s disease were excluded because patients with these mental disorders may experience cognitive deficits and memory impairment that may lead to inaccurate survey responses. Additionally, individuals from countries other than Syria or Jordan were excluded. The students involved in the recruitment process received training in subject interviews, ethical conduct and survey collection from the study PI and also from Dr. Almomani. To ensure diversity within the sample and to improve the generalization of study results, each student involved in data collection in this study was assigned to a specific region within the targeted geographical area. Since the urban area of northern Jordan has many cities/towns, hence different students were assigned data collection from different areas. Additionally, each student was tasked with data collection from a specific number of study participants uniformly distributed among the categories of gender and age. The students described the study objectives in detail to the participants before obtaining consent for participation in the study.

### 2.2 Online surveys

The Arabic versions of the survey questionnaires were uploaded on the UH-REDCap platform for secure survey management and data collection. The students accessed the survey via REDCap link and obtained the participant consent electronically. Participants were provided the option to complete the survey questionnaire either independently or with one-on-one guidance by student researchers. The study team were available to offer any explanations/clarifications to the questions if/when needed by the participants. Upon survey completion, each participant received a $7 (5 Jordanian Dinars (JD)) gift card. Surveys included a sociodemographic questionnaire with general questions on the demographic and socioeconomic circumstances including age, education level, relationship status, number of children, number of family members, health insurance status, employment status, and average monthly income. Educational level was defined as the highest academic qualification achieved. Questions related to prevalence of chronic diseases including diabetes, hypertension, hypothyroidism, asthma, and irritable bowel syndrome were included in the demographic characteristics section, as these measures were reported to be correlated with perceived stress and mental distress (23–26). We evaluated the psychological distress of participants using the Afghan Symptoms Checklist (ASC). The ASC was developed and used in Kabul, Afghanistan, to identify local indicators of psychological distress in conflict and post-conflict situations (27). The Arabic version of the ASC instrument demonstrated excellent reliability in our previous study conducted with Arab American refugees and immigrants who resettled in Houston, TX, USA (28). The ASC is a 22-item instrument that asks about one’s feelings and experience over a 2-weeks period. Each question is asked for the previous 2 weeks. Scores ranged from 22 to 110 derived from response choices ranging from “1” (“never”) to “5” (“everyday”). The ASC questionnaire consists of three interpretable subscales: (1) sadness with social withdrawal and somatic distress, which is indicated by social isolation, crying, lack of appetite, having nightmares, headache, and extreme feeling of hopeless (2) ruminative sadness without social isolation and somatic distress which is indicated by extreme feeling of stress and lack of concentration/focus, and (3) aggression or stress-induced reactivity, which is indicated by quarreling, beating one’s children, and nervousness (29). The instrument demonstrated excellent reliability in this study with a Cronbach’s alpha value of 0.90.

### 2.3 Statistical analysis

Priori power analysis indicated that a sample size of 180/group provides adequate power of at least.90 with an alpha of 0.05 and a medium effect size (*d* = 0.15) to detect the mean difference of the psychometric score between refugee and Jordanian males and females using ANOVA-test. Using descriptive statistics, we conducted sample comparisons between Syrian and Jordanian participants using independent ANOVA test or chi-square test for continuous and categorical variables, respectively. Analysis of covariance (ANCOVA) was used to compare the scores of ASC between Syrians and Jordanians males and females while controlling for participants’ age, employment status, and education level. Multivariable regression model was used to examine the difference in the mean of total ASC score between males and females, Syrian refugee and Jordanian, high education level (college or graduate studies) versus lower education level (high school (HS) or less), employed versus unemployed, and participant age. The overall fit of the model was statistically significant, as indicated by an F-statistic (*F*(5,890) = 15.30, *p* < 0.001), suggesting that the model explains a significant portion of the variance in ASC scores. All the assumptions of the regression model were met. Per our study design, it is clear that the study observations are independent. Further regression diagnostics of residuals showed that no gross departures from linearity and homoscedasticity with reasonable assumption of normality. Our main variables of interest were group (Syrian versus Jordanian), education level, and gender, other variables were selected using forward stepwise variable selection. All analyses were conducted using IBM SPSS (V 29.00, Armonk, NY: IBM Corp). Significance was set at *p* < 0.05.

## 3 Results

A total of 929 subjects (43% Syrian refugees, 56% females) participated in the study. The average age of the participants was 34.91 ± 13.12. The age distribution was comparable between males and females likely wise between Syrian refugees and Jordanians with around 60% of the participants in the age category groups of 18–25 and 26–39 (see Table 1). The difference in education level between Syrian refugees and Jordanians was significant, with more Jordanian having completed higher education than Syrian refugees. A significantly high number of female Syrian refugees did not complete high school education. The disparity in education between Syrians and Jordanians may also be reflected in the monthly income, although the employment rate was comparable between the Syrians and Jordanians, the monthly income was significantly lower among Syrian refugees, in fact 50% of Syrian refugee participants reported monthly income of less than 250 Jordanian Dinar ($352). Around 91% of Syrian refugees did not have health insurance which suggested that they do not have access to governmental health services in Jordan. The prevalence of chronic diseases was comparable between Syrians and Jordanians, importantly, females showed higher prevalence of irritable bowel syndrome than males, particularly Syrian refugee females as compared to refugee males. Both Syrian and Jordanian males reported higher prevalence of cigarette smoking as compared to corresponding females, however, hookah smoking was reported to be comparable among males and females, particularly among Jordanian females who showed similar prevalence of hookah use as Jordanian males (Table 1).

**Table 1:** Sociodemographic characteristics and afghan symptoms checklist mean scores of Syrian and Jordanian participants.

Variable	Categories	Jordanian (C)			Syrian refugee (D)			*p* value^#^
		Male (A) *n* = 231	Female (B) *n* = 296	Total *n* = 527	Male (a) *n* = 179	Female (b) *n* = 223	Total *n* = 402	
		*N* (%) Mean [SD]	*N* (%) Mean [SD]	*N* (%) Mean [SD]	*N* (%) Mean[SD]	*N* (%) Mean [SD]	*N* (%) Mean [SD]	
Age	18–25	**85**_**B**_ (37.6)	80 (27.4)	165 (31.8)	**71**_**b**_ (40.3)	66 (29.9)	137 (34.5)	0.255
	26–39	**83**_**B**_ (36.7)	83 (28.4)	166 (32.1)	7**3**_**b**_ (41.5)	60 (27.1)	133 (33.5)	
	40–59	51 (22.6)	**109**_**A**_ (37.3)	160 (30.9)	30 (17.0)	**86**_**a**_ (38.9)	116 (29.3)	
	60+	7 (3.1)	20 (6.8)	27 (5.2)	2 (1.2)	9 (4.1)	11 (2.7)	
Education	Less than High School	27 (11.9)	**86**_**A**_ (29.2)	113 (21.6)	98 (54.7)	**153**_**a**_ (68.6)	**251**_**C**_ (62.4)	<0.001^***^
	High School	52 (22.9)	71 (24.1)	1**23**_**D**_ (23.6)	25 (14.0)	24 (10.8)	49 (12.2)	
	College	**126**_**B**_ (55.5)	112 (38.0)	**238**_**D**_ (45.6)	**53**_**b**_ (29.6)	45 (20.2)	98 (24.4)	
	Graduate Studies	22 (9.7)	26 (8.8)	**48**_**D**_ (9.2)	3 (1.7)	1 (0.4)	4 (1.0)	
Employment	No	64 (28.4)	**215**_**A**_ (73.9)	279 (54.1)	51 (29.1)	**169**_**a**_ (76.1)	220 (55.4)	0.686
	Yes	**161**_**B**_ (71.6)	76 (26.1)	237 (45.9)	**124**_**b**_ (70.9)	53 (23.9)	177 (44.6)	
Monthly income	<250 JD	27 (11.8)	**63**_**A**_ (21.4)	90 (30.8)	75 (42.1)	**127**_**a**_ (57.2)	**202**_**C**_ (50.5)	<0.001^***^
	250–550 JD	103 (45.2)	130 (44.1)	233 (58.5)	81 (45.5)	84 (37.8)	165 (41.2)	
	551–800 JD	53 (23.2)	61 (20.7)	**114**_**D**_ (83.8)	14 (8.0)	8 (3.6)	22 (5.5)	
	801–1,500 JD	32 (14.0)	33 (11.2)	**65**_**D**_ (90.3)	4 (2.2)	3 (1.4)	7 (1.8)	
	>1,500 JD	13 (5.7)	8 (2.7)	**21**_**D**_ (84.0)	4 (2.2)	0 (0.0)	4 (1.0)	
Relationship	Single	**123**_**B**_ (53.9)	79 (26.8)	**202**_**D**_ (61.4)	**87**_**b**_ (48.6)	40 (17.9)	127 (31.6)	0.221
	Married	97 (42.5)	**186**_**A**_ (63.1)	283 (54.4)	86 (48.0)	**151**_**a**_ (67.7)	237 (59.0)	
	Separated	6 (2.6)	3 (1.0)	9 (52.9)	4 (2.2)	4 (1.8)	8 (2.0)	
	Divorced	1 (0.4)	**8**_**A**_ (2.7)	9 (45.0)	1 (0.6)	**10**_**a**_ (4.5)	11 (2.7)	
	Widowed	1 (0.4)	**19**_**A**_ (6.4)	20 (51.3)	1 (0.6)	**18**_**a**_ (8.1)	19 (4.7)	
Health insurance	No	73_B_ (31.7)	51 (17.2)	124 (23.6)	159 (88.8)	206 (92.4)	**365**_**C**_ (90.8)	<0.001^***^
	Yes	157 (68.3)	**245**_**A**_ (82.8)	**402**_**D**_ (76.4)	20 (11.2)	17 (7.6)	37 (9.2)	
Diabetes	No	217 (93.9)	270 (91.2)	487 (92.4)	169 (94.4)	202 (90.6)	371 (92.3)	0.945
	Yes	14 (6.1)	26 (8.8)	40 (7.6)	10 (5.6)	21 (9.4)	31 (7.7)	
Hypertension	No	**214**_**B**_ (92.6)	252 (85.1)	466 (56.8)	**165**_**b**_ (92.2)	189 (84.8)	354 (88.1)	0.864
	Yes	17 (7.4)	**44**_**A**_ (14.9)	61 (56.0)	14 (7.8)	**34**_**a**_ (15.2)	48 (11.9)	
Hyperthyroidism	No	227_B_ (98.3)	281 (94.9)	508 (96.4)	178 (99.4)	207 (92.8)	385 (95.7)	0.626
	Yes	4 (1.7)	**15**_**A**_ (5.1)	19 (3.6)	1 (0.6)	**16**_**a**_ (7.2)	17 (3.3)	
Asthma	No	223 (96.5)	284 (95.9)	507 (96.2)	172 (96.1)	213 (95.5)	385 (95.8)	0.738
	Yes	8 (3.5)	12 (4.1)	20 (3.8)	7 (3.9)	10 (4.5)	17 (4.2)	
Irritable bowel syndrome	No	204 (88.3)	245 (82.8)	449 (85.2)	156 (87.2)	170 (76.2)	326 (81.1)	0.096
	Yes	27 (11.7)	51 (17.2)	78 (14.8)	23 (12.8)	**53**_**a**_ (23.8)	76 (18.9)	
Cancer	No	231 (100.0)	293 (99.0)	524 (99.4)	178 (99.4)	222 (99.6)	400 (99.5)	0.882
	Yes	0 (0.0)	3 (1.0)	3 (0.6)	1 (0.6)	1 (0.4)	2 (0.5)	
Cigarette smoking	Never	99 (42.9)	**266**_**A**_ (89.9)	365 (69.2)	78 (43.6)	**199**_**a**_ (89.2)	277 (86.9)	0.206
	Former Smoker	**22**_**B**_ (9.5)	2 (0.7)	24 (4.6)	10 (5.6)	0 (0.0)	10 (2.5)	
	Current Smoker	**110**_**B**_ (47.6)	28 (9.5)	138 (26.2)	**91**_**b**_ (50.8)	24 (10.8)	115 (28.6)	
Hookah smoking	Never	154 (66.7)	**231**_**A**_ (78.0)	385 (56.0)	115 (64.2)	**188**_**a**_ (84.3)	303 (75.4)	0.680
	Former Smoker	**17**_**B**_ (7.3)	1 (0.3)	18 (62.1)	**10**_**b**_ (5.6)	1 (0.4)	11 (2.7)	
	Current Smoker	60 (26.0)	64 (21.7)	124 (58.5)	**54**_**b**_ (30.2)	34 (15.3)	88 (21.9)	
Afghan symptoms Checklist (ASC)	Total Score	46.45[1.08]	**51.06**_**A**_ [0.91]	49.04 [16.11]	45.31 [1.28]	**58.22**_**a**_ [1.13]	**52.47**_**C**_ [18.16]	0.002^**^
	Sadness with Social Withdrawal	38.53 [0.93]	**42.55**_**A**_ [0.81]	40.80 [14.14]	37.89 [1.15]	**49.52**_**a**_ [1.01]	**44.34**_**C**_ [16.26]	<0.001^***^
	Ruminative Sadness	5.60 [0.15]	5.97 [0.15]	5.81 [2.45]	5.56 [0.19]	**6.84**_**a**_ [0.16]	**6.27**_**C**_ [2.54]	0.005^**^
	Stress-Induced Reactivity	6.38 [0.19]	**7.86**_**A**_ [0.17]	7.21 [3.00]	5.80 [0.18]	**8.58**_**a**_ [0.21]	7.34 [3.12]	0.512

# *p* value for comparison between groups (Syrian refugee vs Jordanian) using Chi-Square for categorical variables and student t-test for continuous variable.

For each significant pair, the key to the smaller category appears in the category with the larger mean.

Bold faced numbers are significant. ^*^p < 0.05, ^**^p < 0.01, ^***^p < 0.001.

Significance between Jordanian males and females is designated with upper case letters (A, B).

Significance between Syrian refugee males and females is designated with lower case letters (a, b).

Significance between Syrian Refugee and Jordanian groups is designated with upper case letters (C, D).

### 3.1 Psychometric measures

**Figure 1:**
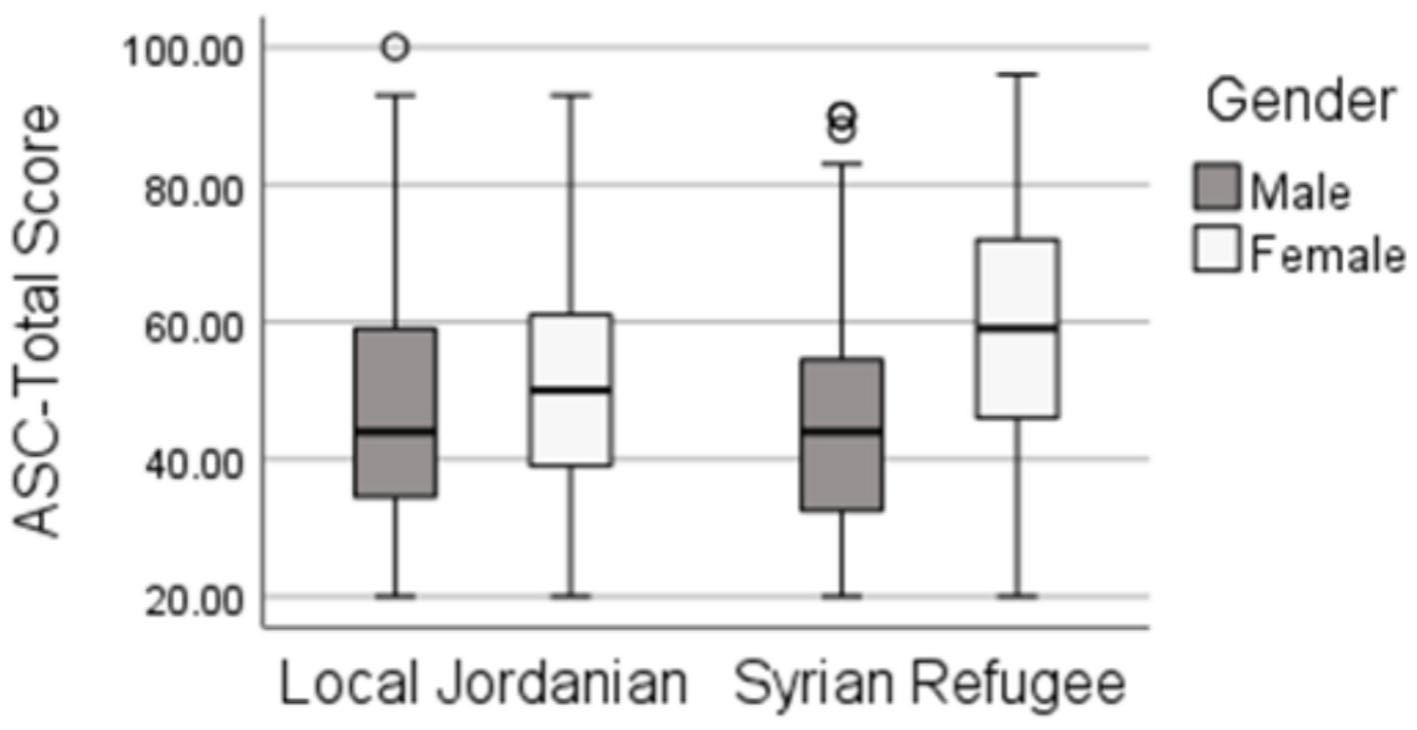
Comparative analysis of mean score for Afghan Symptoms Checklist (ASC) between Jordanian and Syrian refugee males and females.

Mental distress of the participants was evaluated using ASC. Comparative analysis of the ASC total score mean between males and females and Syrian refugee and Jordanian showed that Syrian refugee females exhibited the highest score as compared to Syrian refugee males as well as to Jordanian males and females (Syrian refugee females: 58.22 ± 1.13; Syrian refugee males: 45.31 ± 1.28; Jordanian females: 51.06 ± 0.91; Jordanian males: 46.45 ± 1.08, *p* = 0.002) (see Figure 1). Syrian refugee females also exhibited higher scores in with social withdrawal subscale as well as in ASC-ruminative sadness subscale as compared to the other groups. They also exhibited higher scores in ASC-stress induced reactivity as compared to Syrian males (see Table 1). Jordanian females exhibited higher scores in ASC-sadness with social withdrawal subscale as well as in ASC-stress induced reactivity subscale as compared to Jordanian males (see Table 1).

### 3.2 Predictors of mental distress

We employed a multivariable regression model to examine the difference in the mean of total ASC score between Syrian refugee and Jordanian, males and females, individuals with high education level versus individuals with education level (high school (HS) or less), employed versus unemployed, while controlling for age (Table 2). Regression analysis showed that the estimated difference in the mean of ASC score between males and females was 7.42 (*p* < 0.001), and the estimated mean difference between Syrian refugees and Jordanians is 2.76 (*p* = 0.019) (Table 2). Interestingly, the estimated mean of ASC scores for individuals with high education level was decreased by 4.18 as compared to individuals who had lower education level (high school or less), *p* < 0.001.

**Table 2:** Multivariable linear regression model for predictors of Afghan Symptoms Checklist (ASC) scores among Syrian refugees and Jordanian males and females.

Variable	Estimate (*β*)	Std. Error	*t*	*p*-value	95.0% CI
Gender (ref. male)					
Female	7.420	1.280	5.798	<0.001^***^	4.909, 9.932
Group (ref. Jordanian)					
Syrian Refugee	2.759	1.173	2.352	0.019^**^	0.457, 5.060
Education (ref. HS or less)					
College or graduate studies	−4.180	1.231	−3.397	<0.001^***^	−6.595, −1.765
Employment (ref. unemployed)					
Employed	0.454	1.251	0.363	0.717	−2.002, 2.910
Age					
Age	0.006	0.045	0.132	0.895	−0.081, 0.093

^*^Significant at *p* < 0.05, ^**^Significant at *p* < 0.01, ^***^Significant at *p* < 0.001.

## 4 Discussion

This cross sectional study assessed mental health status of Syrian refugees who are resettled in the northern part of Jordan and the association between education level and mental health outcomes also were examined in this vulnerable population. According to the data obtained in the present study, Syrian refugee females exhibited significantly higher scores in the Afghan Symptoms Checklist (ASC) questionnaire when compared to the data reported by Syrian refugee males and also as compared to local Jordanian males and females, an indication of elevated mental distress. This is not surprising considering the frequently reported prevalence of elevated mental distress and high mental disorders among refugees when compared to the host communities (30, 31), particularly among female refugees who reportedly exhibit significantly higher mental health problems than male refugees (28, 31). The reasons behind high mental distress especially among female refugees are complex and multifactorial, ranging from enduring the experiences of acute stressors of war, to contextual pre-and post-migration sociopolitical stressors such as financial vulnerability, food insecurity, housing challenges, and other cultural integration barriers (31). Importantly, in the present study, low education level was observed to be highly predictive of elevated mental distress as revealed by logistic regression analysis. Present data also showed that employment rate was comparable between Jordanian and Syrian refugees, however the reported monthly income was significantly lower among Syrian refugees when compared to Jordanian adults (Table 1). These are important observations which are critical for a variety of reasons. For example, education level and employment are interconnected, and are commonly considered a predictor of quality of life. In fact, refugees with post-secondary education, both men and women, are reportedly more financially stable than those with less education (32). Additionally, poverty and economic adversity are well-known risk factors for adverse mental health outcomes among refugees (33). This may be attributed to the fact that refugees face multiple barriers in gaining employment in the host countries due to visa restrictions, communication barriers (especially when resettling in western countries), lack of education, cultural competency issues, and discrimination, and racism. Refugees are also often underemployed (i.e., employed in positions below their qualification level or skills) as compared to local host communities (34). In the case of Syrian refugees in Jordan, according to the agreement signed in 2016 between the European Union (EU) and Jordan, the Jordanian government allowed Syrian refugees a formal access to the labor market (35, 36, 50), thus granting them work permits in certain sectors, although there still remains a disproportionate level of employment of migrant workers (37).

In the present study, the difference in the education level between Syrian refugees and Jordanian adults was significant, with more Jordanians reported having completed higher education than Syrian refugees (Table 1). In particular, Syrian refugee females reported low education level which was highly associated with mental distress (Table 2). This is consistent with the latest statistics which shows that the education inequities between Syrian refugees as compared to Jordanians are extreme: with 24% of Jordanian students receiving higher education each year, and only 3% of Syrian refugee students enrolled in Jordanian universities, and even fewer of them reportedly finishing higher education degrees (51). Education is considered a foundational component for long-term development of displaced populations, therefore, it is increasingly integrated into humanitarian relief efforts (49), however, higher education for refugees still remains low on the agenda and is often perceived a luxury with primary or secondary education receiving more attention (38). Consequently, higher education opportunities remain limited for refugees globally and for Jordan-based Syrian refugees in particular (22, 39). The limitation of higher education opportunities for Syrian refugees is further attributed to the fragile socio-economic situation of the kingdom and to the unstable geopolitical environment, especially due to the ongoing Israel-Hamas war in the Middle East, thus shifting the global attention to this highly precarious conflict. Hence, the economic slowdown and the major decline in international funding has drastically reduced the number of scholarships available for refugees, reducing tuition affordability for Syrian refugee students in Jordan. Moreover, scholarships and post-graduate job restrictions limit Syrians to only a certain subject matter areas, delimiting them from prestigious fields of medicine, pharmacy and engineering, thus limiting opportunity and agency for young Syrians when deciding their future careers (39).

**Figure 2:**
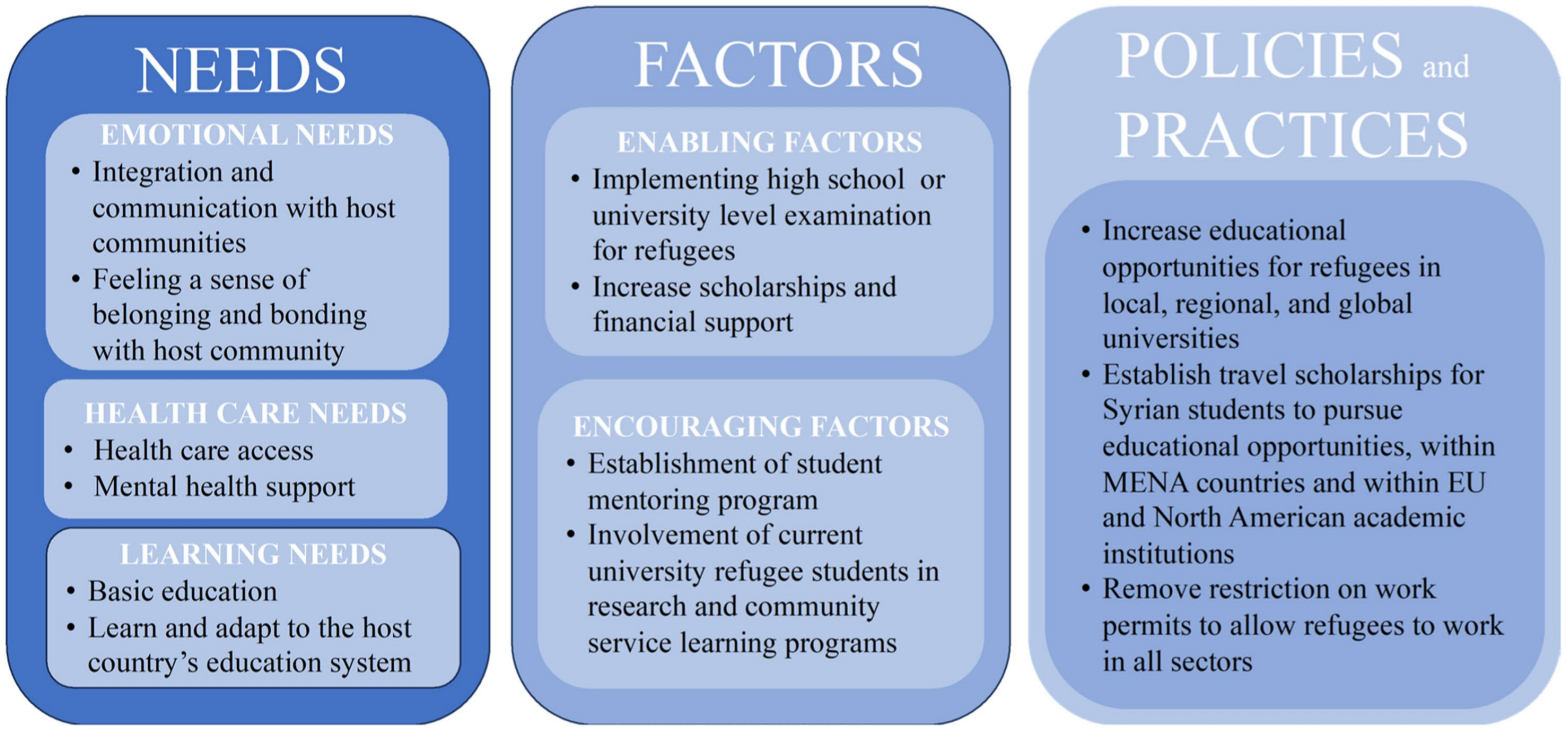
Holistic model to support refugee enrollment in higher education.

Drawing on previous evidence and key factors identified in the present study, we propose a holistic model to support better mental health outcomes for Syrian refugees in Jordan (Figure 2). Since we have observed a significant association between education, socioeconomic status and mental distress, our model considers refugee educational integration is central to the overall quality of life. Further, our model also suggests that the social and emotional needs of refugees stemming from experiences of trauma, grief and stress of resettlement also are critical and must be addressed in a timely manner. The model we propose depicts how the interplay of refugee needs, enabling and encouraging factors, may inform the policies and practices consequently supporting integration of refugees in the host country higher education system. Integration of refugee students in higher education can be achieved if their learning, social and emotional needs are appropriately met. Refugees must familiarize themselves with the host country’s education system, and learn to adapt within the host communities, to promote a sense of belonging and bonding with the host community. At the same time, the host communities also must actively engage with the refugee students to actively participate in their educational empowerment. Overall, the Jordanians have demonstrated an exemplary humanitarian stance toward Syrian refugees (40), this combined with the historically similar cultural and geographical relationship between the two countries (40) may further facilitate true societal transformation. Furthermore, efforts to help cope with loss, grief as well as separation and/or trauma can be achieved by providing health care access and mental health support for refugees. Different enabling and encouraging factors can effectively enhance the integration process. According to previous evidence (22, 38, 39), we propose the following enabling factors to enhance refugee enrollment in higher education; First, enable eligible Syrian refugee students to complete high-school graduation examinations or university level exams thus preserving their academic continuity. Second, increase scholarships and financial support. We also propose the following encouraging factors; (1) establishment of student mentoring program, and (2) involvement of current university refugee students in research and community service programs. For example, data collection in the present study was conducted by Jordan University of Science and Technology (JUST) students, some of whom were Syrian refugee students enrolled in JUST Master’s program, who may serve as an excellent role models for refugee communities. According to previous studies (37, 38), the proposed polices and practices involve; (1) Increasing educational opportunities for refugees in local, regional, and global universities. (2) Establish travel scholarships for Syrian refugee students to pursue educational opportunities, within MENA countries and within EU and North American academic institutions. (3) Remove restriction on work permits to allow refugees to work in all sectors. The proposed programs and policies implemented in this holistic model target several core issues which might help mitigate challenges faced by refugees. Access of higher educational opportunities to refugees and improving their socioeconomic status may also be effective in buffering mental distress and building resiliency among displaced communities. Increasing the opportunities and accessibility to higher education will enable refugees achieve financial freedom which can mitigate their mental distress and improve their quality of life.

Finally, elevating refugee enrollment in higher education by fully incorporating UNHCR’s directive in Jordan is crucial for development. Several important factors including financial impediments, infrastructural deficits, bureaucratic barriers, and socio-cultural deterrents have already been identified, which appear as road blocks in achieving this goal (41). A multipronged unified approach via engagement of local NGOs (Save the Children, Mercy Corps) and local community leaders is needed for a meaningful transformation in the Jordanian society. This approach will most likely mitigate the disconnect often noted among donors, local governments, and educational institutions, which creates inefficient and duplicated efforts. Legislative amendments are also needed to provide full access for refugees to higher education, which might require amendment in admission policies and fee structures to increase accessibility to higher education for the refugees. Thus, a multi-layered solution is desired including instituting targeted scholarship programs, institutional capacity building initiatives such as the one offered by the National Institutes of Health Fogarty programs in the United States, maximizing digital educational platforms, and fostering global educational partnerships. Thus, by strategically enhancing and providing global higher education opportunities for refugees, Jordan may create a highly skilled workforce while upholding its long history of continued commitment to inclusivity, and equity.

### 4.1 Limitations

The results of this study are important and intriguing, but several methodological and situational limitations were noted which are discussed here. First, logistic regression analysis indicated that low education level is highly predictive of elevated mental distress. However, the role of other confounding factors, such as discrimination, family size, social integration, etc. as a risk factor to mental distress cannot be excluded, hence this remains as one of the study limitations. Second, although Jordan-based Syrian refugees share common religious and linguistic background with the local Jordanian host communities, yet the cultural, social and traditional nuances between the groups are often too stark. Previous evidence suggests that although refugee and host populations residing in low-income and middle-income neighboring regions (42), often share cultural, religious, and linguistic backgrounds (43, 44), yet, the adaptation is often complex (45), which western researchers often fail to consider in data analysis or interpretation (46). Finally, this is a descriptive cross-sectional study which was aimed to characterize the status of mental health of Syrian refugees as compared to local Jordanian sample, the other goal was to examine the predictive variables of mental health focusing on education level and gender. Although the study provided significant insight into the impact of these variables on the mental health of Syrian refugees resettled in Jordan, causal relationships, which are often multi-factorial and complex, cannot be derived from this cross-sectional analysis.

## Data Availability

The raw data supporting the conclusions of this article will be made available by the authors, without undue reservation.
